# Kinsenoside Protects Against Radiation-Induced Liver Fibrosis *via* Downregulating Connective Tissue Growth Factor Through TGF-*β*1 Signaling

**DOI:** 10.3389/fphar.2022.808576

**Published:** 2022-01-21

**Authors:** Xiaoqi Nie, Qianqian Yu, Long Li, Minxiao Yi, Bili Wu, Yongbiao Huang, Yonghui Zhang, Hu Han, Xianglin Yuan

**Affiliations:** ^1^ Department of Oncology, Tongji Hospital, Huazhong University of Science and Technology, Wuhan, China; ^2^ Department of Dermatology, Tongji Hospital, Huazhong University of Science and Technology, Wuhan, China; ^3^ School of Pharmacy, Tongji Medical College, Huazhong University of Science and Technology, Wuhan, China

**Keywords:** radiation-induced liver fibrosis, kinsenoside, hepatic stellate cells, transforming growth factor-β1, connective tissue growth factor

## Abstract

Radiation-induced liver fibrosis (RILF) is a serious complication of the radiotherapy of liver cancer, which lacks effective prevention and treatment measures. Kinsenoside (KD) is a monomeric glycoside isolated from *Anoectochilus roxburghii*, which has been reported to show protective effect on the early progression of liver fibrosis. However, the role of KD in affecting RILF remains unknown. Here, we found that KD alleviated RILF via downregulating connective tissue growth factor (CTGF) through TGF-β1 signaling. Sprague-Dawley rats were administered with 20 mg/kg KD per day for 8 weeks after a single 30Gy irradiation on the right part of liver, and tumor-bearing nude mice were administered with 30 mg/kg KD per day after a single fraction of 10Gy on the tumor inoculation site. Twenty-four weeks postirradiation, we found that the administration of KD after irradiation resulted in decreased expression of *α*-SMA and fibronectin in the liver tissue while had no adverse effect on the tumor radiotherapy. Besides, KD inhibited the activation of hepatic stellate cells (HSCs) postirradiation via targeting CTGF as indicated by the transcriptome sequencing. Results of the pathway enrichment and immunohistochemistry suggested that KD reduced the expression of TGF-*β*1 protein after radiotherapy, and exogenous TGF-*β*1 induced HSCs to produce *α*-SMA and other fibrosis-related proteins. The content of activated TGF-*β*1 in the supernatant decreased after treatment with KD. In addition, KD inhibited the expression of the fibrosis-related proteins by regulating the TGF-*β*1/Smad/CTGF pathway, resulting in the intervention of liver fibrosis. In conclusion, this study revealed that KD alleviated RILF through the regulation of TGF*β*1/Smad/CTGF pathway with no side effects on the tumor therapy. KD, in combination with blocking the TGF-*β*1 pathway and CTGF molecule or not, may become the innovative and effective treatment for RILF.

## Introduction

Radiotherapy is an important treatment method for liver cancer. However, the damage to normal tissues caused by radiotherapy often restricts the efficacy of radiotherapy, and the delayed organ damage such as the radiation-induced liver fibrosis (RILF) is inevitable and even lethal in some cases (Du et al., 2010; Han et al., 2019). Radiation-induced liver injury is generally divided into the subacute radiation-induced liver disease stage and the advanced RILF stage (Liang et al., 2018). The loss of liver parenchymal cells, the destruction of liver lobule structure and the hyperplasia of fibrous connective tissue are the main histological characteristics of the RILF (Lee and Friedman 2011; Kim and Jung 2017). The radiation-induced tissue fibrosis was formerly considered to be inevitable and irreversible, while it is currently believed that the radiation-induced fibrosis is caused by the dynamic interaction between multiple cell types in specific organs, suggesting that the RILF may be regulable (Du et al., 2010; Hu et al., 2018). However, the potential regulatory mechanism needs to be further explored. As the number of long-term surviving patients largely increased with the improvement of the therapeutic effect for liver cancer, one of the main directions of the radiobiological research is to reduce and treat the late liver fibrosis caused by the radiotherapy (Jung et al., 2013; Atta 2015; Cheng et al., 2015).

*Anoectochilus roxburghii* (*A. roxburghii*), a traditional Chinese herbal plant, has a variety of pharmacological effects, including anti-obesity, anti-hyperglycemia, anti-osteoporosis, and so on, among which the liver-protecting effect shows significant effect in clinical use (Ye et al., 2017). Kinsenoside (3-(R)-3-*β*-D-Glucopyranosyloxybutanolide, KD) is a biologically active compound isolated and extracted from *A. roxburghii* which has been reported to have the anti-hyperglycemic and anti-hyperlipidemic effects, and to alleviate the acute inflammation. In addition, KD has shown a protective effect on the liver in the mice and the patients with liver disease, but the mechanism is not yet clearly understood (Tullius et al., 2014; Zarzycka et al., 2014; Zhang et al., 2014; Qi et al., 2018; Ming et al., 2021). Up to now, there is no research focusing on the roles of KD playing in the RILF.

When activated, myofibroblasts are the most important collagen-producing cells in the fibrosis process, and it is generally believed that hepatic stellate cells (HSCs) are the main source of collagen-producing fibroblasts in the cirrhotic liver (Wei et al., 2013). Studies have shown that HSCs are the critical effector cells in the process of the RILF (Oakley et al., 2005; Hasan et al., 2017; Dewidar et al., 2019b). In the development of the RILF, HSCs are activated and continue to proliferate, which leads to the imbalance and interaction of various fibrosis-related cytokines, ultimately resulting in the accumulation of the extracellular matrix (ECM) (Wang et al., 2013; Chen et al., 2017; Yuan et al., 2019a).

The transforming growth factor-*β* (TGF-*β*) signaling pathway family is the core member that maintains the dynamic balance of tissues and organs, and plays a vital role in regulating cell proliferation, differentiation, migration or death (Derynck and Budi 2019; Dituri et al., 2019; Katsuno et al., 2019). As reported, TGF-*β* is not only a key regulator of liver pathophysiology, but also one of the most important pro-fibrotic cytokines in the process of liver fibrosis (Xu et al., 2016; Wu et al., 2018b; Mu et al., 2018; Dewidar et al., 2019a; Dewidar et al., 2019b).

Based on these existing research results, we studied whether KD could protect the liver after radiation and interfere with the RILF. In addition, to further clarify the mechanism through which KD affected the RILF, we tested the effect of KD on the HSCs and screened the key target molecules and signaling pathways affected by KD.

## Materials and Methods

### Ethics Statement

Male Sprague-Dawley (SD, Experimental Animal Center of Hubei Province, China) rats aged 7–8 weeks and Balb/c nude mice (Hunan SJA Laboratory Animal Co., Ltd., Hunan, China) aged 4 weeks were housed in the specific pathogen-free breeding system. All rats and mice were randomly grouped and adaptively fed for a week. All the experimental designs and procedures were conducted in accordance with the ARRIVE guidelines and the National Institutes of Health guide for the care and use of Laboratory animals (NIH Publications No. 8023, revised 1978).

### Irradiation and Kinsenoside Treatment of SD Rats

The SD rats were divided into four groups by randomization (*n* = 5 per group): Control group (Con), only KD group (KD), irradiation group (IR) and irradiation treated with KD group (IR + KD). The radiation dose was a single 30Gy for the irradiation groups, and the irradiation field was 2.5 × 2.5 cm in the right part of the liver (RS2000 X-ray Biological Research Irradiator, 25 mA, 160 kV; Rad Source Technologies Inc., Suwanee, GA). The KD powder was obtained from the School of Pharmacy, Tongji Medical College, Huazhong University of Science and Technology, and the KD solution was made by dissolving the powder in the pure water. The KD solution was given 20 mg/kg per day by oral gavage, while the Con group and IR group were fed with pure water at the same time. The gavage feeding time for the irradiation groups was 8 weeks postirradiation. The animals used to observe the pathological results were euthanized at the 24th week after irradiation.

### Irradiation and Transplantation Tumor Experiment of Nude Mice

The hepatocellular carcinoma cells (HepG2) were injected into the subcutaneous tissue under the left upper limb of nude mice. The diameters of the xenograft tumors were measured and the mice weighed every 3 days. When the mean size of the xenograft tumors reached 85–120 mm^3^, the tumor-bearing nude mice were divided into four groups by randomization (*n* = 7 per group): Con group, KD group, IR group and IR + KD group. The radiation dose was a single 10Gy for the irradiation groups, and the irradiation fields were the tumor inoculation sites. The KD solution was given 30 mg/kg per day by oral gavage immediately postirradiation. The gavage feeding time was 12 days until the nude mice were euthanized.

### Liver Histology

At the 24th week postirradiation, the rats were euthanized and the liver tissues were cut into pieces. Part of the liver tissues were immersed in 4% paraformaldehyde for paraffin embedding. The liver sections were stained with Masson’s trichrome and HE staining (Aspen Biological, Wuhan, China). The Masson’s-stained sections were used to assess the degree of liver fibrosis using the METAVIR scoring method. All the scoring work was carried out by two independent senior pathologists that were blinded to the experiment.

### Immunohistochemistry and Immunofluorescence

For the immunohistochemistry (IHC) of the liver tissues, the primary antibodies were TGF-*β*1 (1:200; Cell Signaling Technology Inc., Danvers, MA) and *α*-smooth muscle actin (*α*-SMA) (1:200; Servicebio Technology, Wuhan, China). For the immunofluorescence of the liver tissues and HSCs, the primary antibodies were collagen (1:200; BOSTER Biological Technology, Wuhan, China) and *α*-SMA (1:200; Servicebio Technology, Wuhan, China).

The IHC scores were based on the staining intensity and positive-stained cells. Five fields of each slice were randomly selected and the average value was the final IHC score. All the scoring work was carried out by two independent senior pathologists that were blinded to the experiment.

### Western Blot Analysis

Liver tissues (30–100 mg) were homogenized and the protein liquid was extracted. The proteins were separated and transferred to the polyvinylidene difluoride (PVDF) membranes (Millipore, Billerica, MA). The PVDF membranes were incubated with primary antibody overnight at 4°C. The antibodies included: *α*-SMA (1:1,000; Servicebio Technology, Wuhan, China), fibronectin (FN), collagen I and connective tissue growth factor (CTGF) (1:1,000; BOSTER Biological Technology, Wuhan, China), TGF-*β*1 and Smad2/3 and P-Smad2/3 (1:1,000; Cell Signaling Technology Inc., Danvers, MA), GAPDH and *β*-actin (1:1,000; Aspen Biological, Wuhan, China). Then, the PVDF membranes were incubated with the anti-rabbit IgG secondary antibody (1:6,000; Aspen Biological, Wuhan, China) at room temperature for 1 hour. Finally, the proteins were detected with the Super Signal West Pico plus Chemiluminescent Substrate (Thermo Fisher Scientific, Waltham, MA).

### Hydroxyproline Content Assay

The content of hydroxyproline in the liver tissues was detected by the hydroxyproline assay kit (Nanjing Jiancheng Bioengineering Institute, Nanjing, China). All the steps were implemented strictly following the instructions.

### Cell Culture and Treatment

The immortalized hepatic rat stellate cell line (HSC-T6) was kindly provided by Procell Life Science Technology Co., Ltd. (Wuhan, China). The HSCs were cultured in Dulbecco’s Modified Eagle’s Medium (DMEM) supplemented with 10% fetal bovine serum (FBS, Gibco). The culture incubator for the cells was 37°C with constant temperature, constant humidity, and 5% CO_2_. The HSCs were seeded in the 6-well plates and cultured for about 24 h before treated with different concentrations of KD and TGF-*β*1, respectively. After that, cells were cultured for 24 h until further detection.

### Real-Time Fluorescence Quantitative PCR

The RNA of the cells was extracted with TRIzol solution, then the concentration of RNA in each group was determined. The cDNA was obtained according to the reverse transcription cDNA synthesis kit (Thermo, United States). The relative expression of genes was finally calculated according to the SYBR Green PCR kit (Thermo, United States) and the 2^−ΔΔCt^ method.

### High Throughput mRNA Sequencing

Total RNA was extracted with the TRIzol Reagent kit (Invitrogen), quantified and qualified with NanoDrop (Thermo Fisher Scientific Inc.) and Agilent 2,100 Bioanalyzer (Agilent Technologies). Next generation sequencing library preparation was constructed using 1 μg total RNA with RIN value above 6.5 in accordance with the manufacturer’s instructions. The Poly(A) mRNA Magnetic Isolation Module or rRNA Removal Kit was used to isolate the poly(A) mRNA.The mRNA fragmentation and priming was performed using First Strand Synthesis Reaction Buffer and Random Primers. cDNA was synthesized using the ProtoScript II Reverse Transcriptase and the Second Strand Synthesis Enzyme Mix, followed by purification by beads. The End Prep Enzyme Mix and the T-A ligation was used to repair cDNA ends, add a dA-tailing, and add adaptors to both ends. DNA fragments around 420 bp were then recovered in the size selection. The P5 and P7 primers were used for sample amplification by PCR for 13 cycles. The PCR products were then cleaned up, validated and quantified.

The Illumina HiSeq instrument (Illumina, San Diego, CA, United States) was then used in accordance with the manufacturer’s instructions. The libraries were multiplexed and loaded. The 2 × 150 bp paired-end (PE) configuration was used for sequencing. The HiSeq Control Software (HCS) + OLB + GAPipeline-1.6 (Illumina) was used for image analysis and base calling. GENEWIZ was used to process and analyze the sequences.

### Cell Cycle Detection

The cells were digested and the cell suspension was centrifuged. The cells were resuspended in 1ml of pre-cooled 70% ethanol solution and placed in the −20°C refrigerator for more than 12 h. After the fixation, the cells were washed twice and treated in accordance with the requirements of the cell cycle detection kit (Servicebio Technology, Wuhan, China).

### Enzyme Linked Immunosorbent Assay

The cell culture supernatant was separated for testing, and the TGF-*β*1 content in the supernatant was detected according to the enzyme linked immunosorbent assay (ELISA) kit (ELK Biotechnology, Wuhan, China) instructions.

### Statistical Analysis

In this study, GraphPad Prism software was used to perform statistical data analysis. The results were expressed as mean ± standard error of mean (SEM). Student’s *t* test was used to analyze continuous variable data. *p < 0.05, **p < 0.01, ***p < 0.001, ****p < 0.0001. *p* < 0.05 was statistically significant.

## Results

### Kinsenoside Attenuates Radiation-Induced Liver Fibrosis in the Rat Model

**FIGURE 1:**
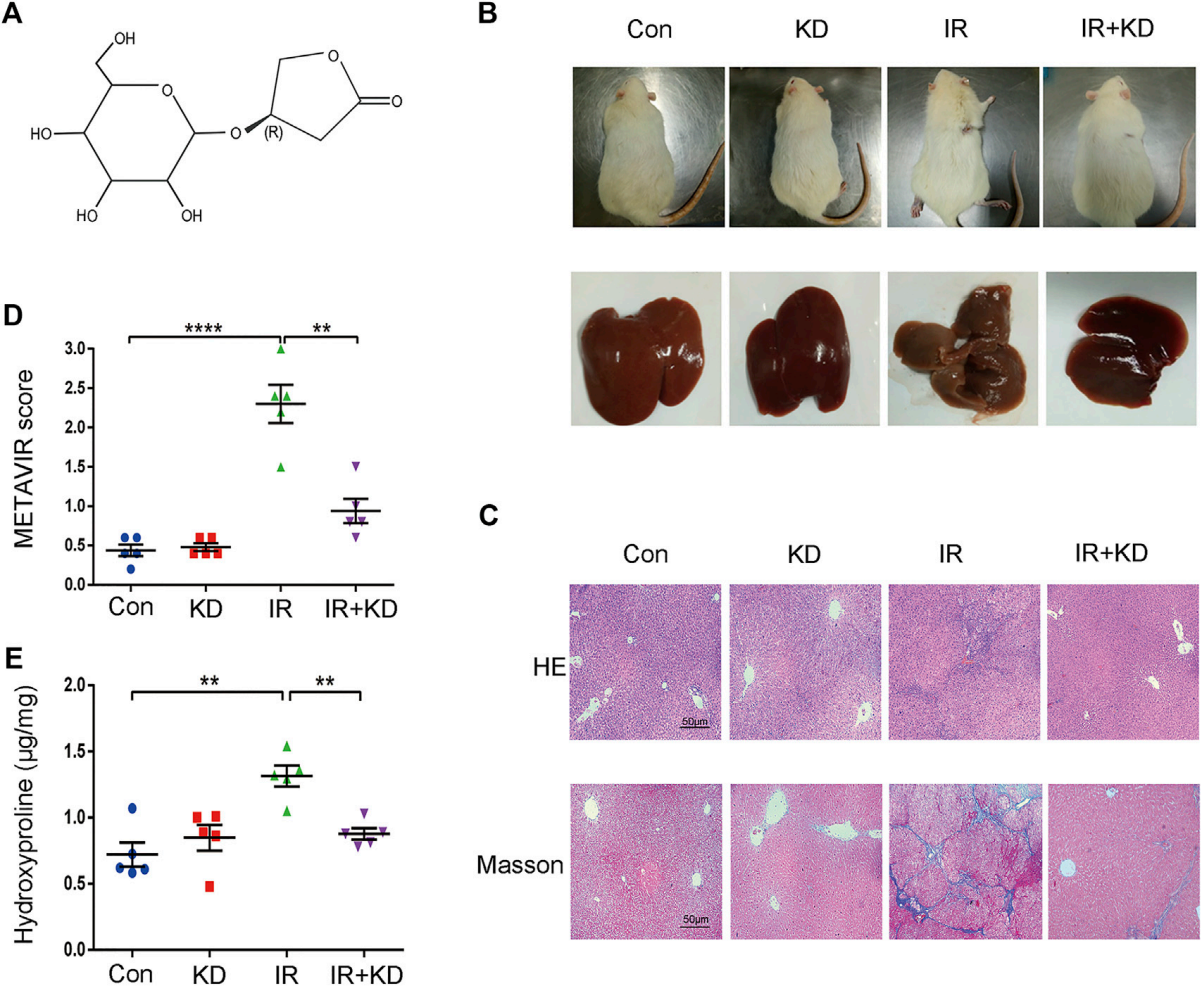
KD alleviates the RILF in the rat model **(A)** Chemical structure of KD **(B)** Photograph of irradiated areas and part of the livers of the rats. The liver was yellowish and rough in the IR group, while in the other groups the livers were ruddy and smooth **(C)** RILF was assessed through the H&E and Masson’s trichrome staining of representative liver slices. In the IR group, blue stained collagen deposited in liver tissues, while KD treatment significantly attenuated the radiation induced injury **(D)** METAVIR scores for grading the liver fibrosis. KD treatment attenuated the degree of fibrosis **(E)** The content of hydroxyproline in the liver tissues. Hydroxyproline content increased after irradiation and decreased significantly in the IR + KD group. For all results in this figure, original magnification, ×100. Mean ± SEM. n. s. denotes not significant; **p < 0.01, ***p < 0.001, ****p < 0.0001.

In order to investigate the effect of KD on the RILF, we established the animal model of the RILF. 24 weeks after irradiation, the partial hair loss and ulceration appeared in the irradiated area. Besides, the liver was yellowish and the surface of the liver was rough and grainy in the IR group, while in the IR + KD group the liver was ruddy and smooth (Figure 1B). Next, the pathological analysis results of the HE and Masson staining showed that the rats suffered RILF from irradiation, and the normal liver lobule structure of the rat liver was destroyed, with the central vein even collapsed. There was obvious fibrous connective tissue deposition in the liver, and the portal area was often more obvious, while KD could attenuate RILF. KD administration significantly reduced the deposition of collagen fibers, and the damage to the liver structure and central veins was also reduced (Figure 1C). METAVIR fibrosis score and hydroxyproline content were important indicators for judging the degree of liver fibrosis (Bedossa and Poynard 1996). The liver fibrosis score of the IR group was significantly higher than that of the Con group, while the IR + KD group score decreased (Figures 1D,E), indicating that KD administration after irradiation attenuated liver tissue fibrosis caused by radiation.

### Kinsenoside Inhibits Expression of the Fibrosis-Related Proteins in the Rat Model

**FIGURE 2:**
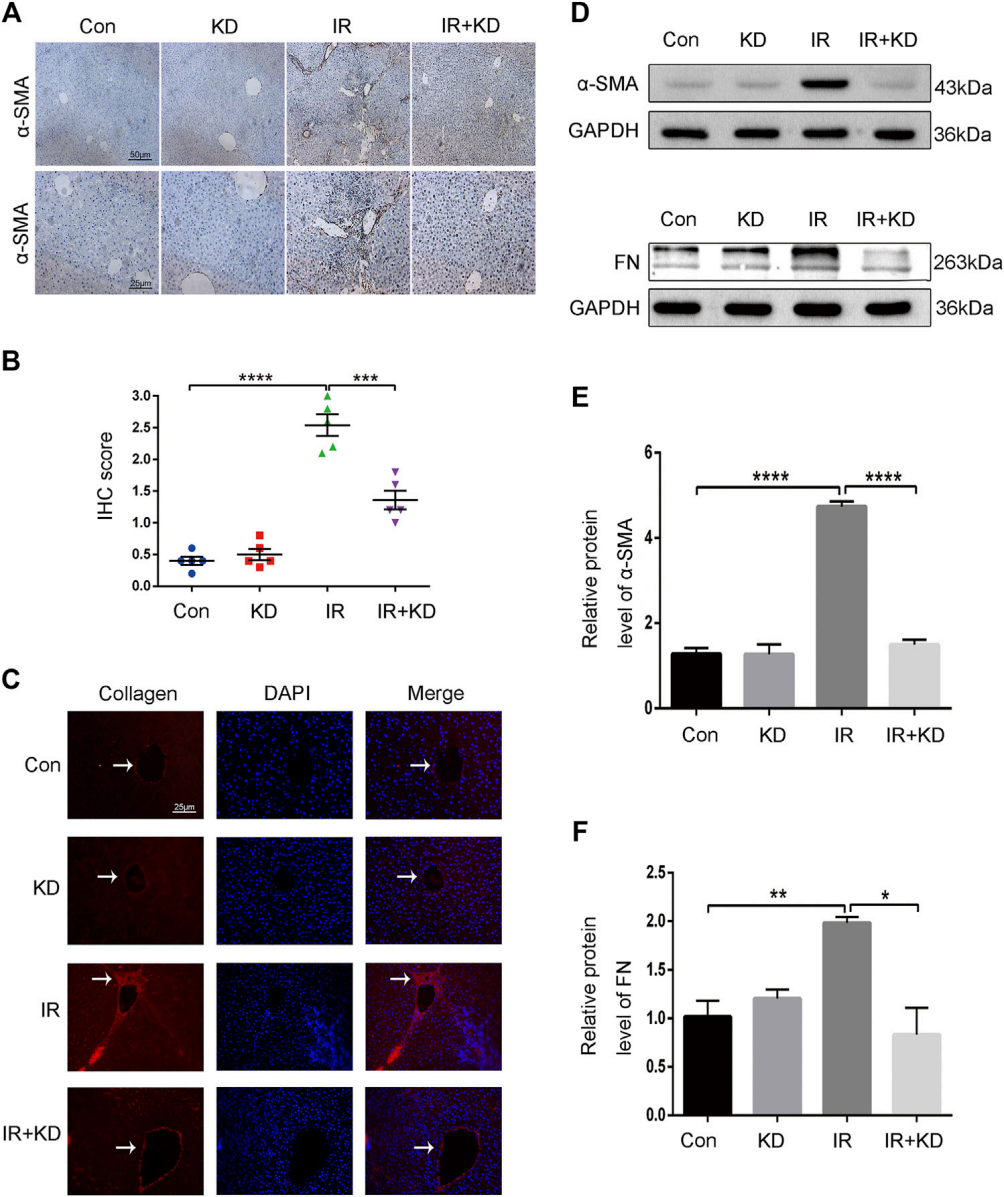
KD inhibits the expression of the fibrosis-related proteins in the rat model **(A)** The results of the IHC staining displayed the expression of *α*-SMA of the liver histology slices. KD significantly reduced the expression of brown stained *α*-SMA after irradiation **(B)** The IHC scores for grading the expression of *α*-SMA **(C)** Expression and localization of collagen in the liver tissue. The red stain represented collagen, which indicated that collagen expression in the IR + KD group was significantly lower than that in the IR group **(D-F)** The results of the western blots showed the expression of *α*-SMA and FN proteins in the liver tissue. The administration of KD after irradiation resulted in decreased expression of *a*-SMA and FN. For all results in this figure, original magnification, ×100 and ×200. Mean ± SEM. n. s. denotes not significant; *p < 0.05, **p < 0.01, ***p < 0.001, ****p < 0.0001.

The expression level of *α*-SMA is an important sign of HSC activation, and it is also one of the important indicators to judge the degree of liver fibrosis (Liu et al., 2015). Besides, FN was an important ECM regulatory component in the fibrosis process related to HSCs activation (Zollinger and Smith 2017; Klingberg et al., 2018). At the 24th week postirradiation, the liver tissues of the rats in each group were subjected to *α*-SMA immunohistochemistry. The expression of *α*-SMA increased in the liver portal area of the rats in the IR group, and the expression level of *a*-SMA in the IR + KD group was significantly lower than that of the IR group (Figures 2A,B). We performed immunofluorescence staining of liver tissue collagen. The results showed that the expression of collagen in the liver portal area increased in the IR group, and was significantly reduced in the IR + KD group (Figure 2C), indicating that the degree of fibrosis was significantly reduced after the administration of KD. Furthermore, the results of Western Blot analysis showed that administration of KD after irradiation resulted in decreased expression of *α*-SMA (Figures 2D,E) and FN (Figures 2D,F) in liver tissue. Based on these results, KD inhibited the expression of the fibrosis-related proteins, and alleviated RILF in the rat model.

### Kinsenoside Exerts No Adverse Effects on the Radiosensitivity of Liver Cancer in the Mouse Model

**FIGURE 3:**
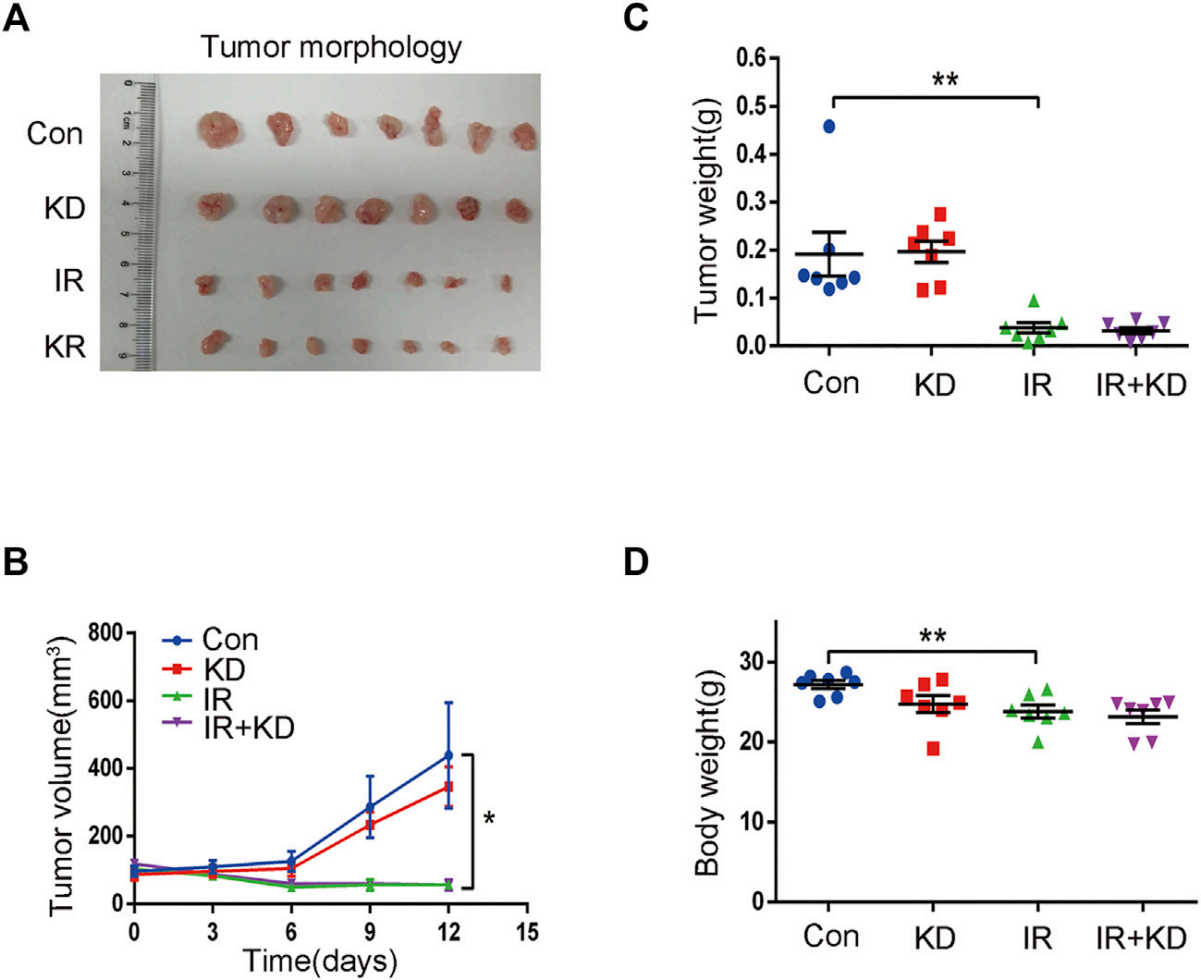
Treatment with KD does not alter the radiosensitivity of liver cancer in the mouse model **(A)** In the IR group and the IR + KD group, the tumor size significantly reduced compared with the Con group and the KD group **(B)** The tumor volume-time curve showed that the tumor volume of the irradiated groups increased slower than the control groups, and there was no statistical difference in the tumor volume between the IR group and the IR + KD group **(C)** Compared with the control group, the tumor weight was significantly reduced after irradiation, but there was no statistical difference in tumor weights between the IR group and the IR + KD group **(D)** There was no statistical difference in the body weights of nude mice between the IR group and the IR + KD group. For all results in this figure, mean ± SEM. n. s. denotes not significant; *p < 0.05, **p < 0.01.

In order to test whether KD reduced the efficacy of radiotherapy for liver cancer, we tested the effects of KD after the inoculation of liver cancer cells (HepG2) in the nude mice. The tumor size in the IR and IR + KD groups decreased significantly after irradiation, and the tumor volume-time curve showed that the tumor volume in the irradiated groups increased slowly compared to the control groups, while there was no statistical difference in the tumor volume between the IR group and IR + KD group (Figures 3A,B).

In addition, the tumor weight of the IR group and IR + KD group was significantly lower than those of the control groups, but there was no statistical difference in the tumor weight between IR group and IR + KD group (Figure 3C). Furthermore, the body weight of nude mice in each group was determined. The body weight after irradiation was reduced compared with the control groups, but there was no difference between the two irradiation groups (Figure 3D). Overall, these above results comprehensively showed that the administration of KD after irradiation did not affect the efficacy of tumor radiotherapy, suggesting that KD did not affect the curative effect of radiotherapy on tumors while inhibiting RILF.

### Kinsenoside Inhibits Cell Proliferation of Hepatic Stellate Cells and Activation of Fibrosis-Related Proteins

**FIGURE 4:**
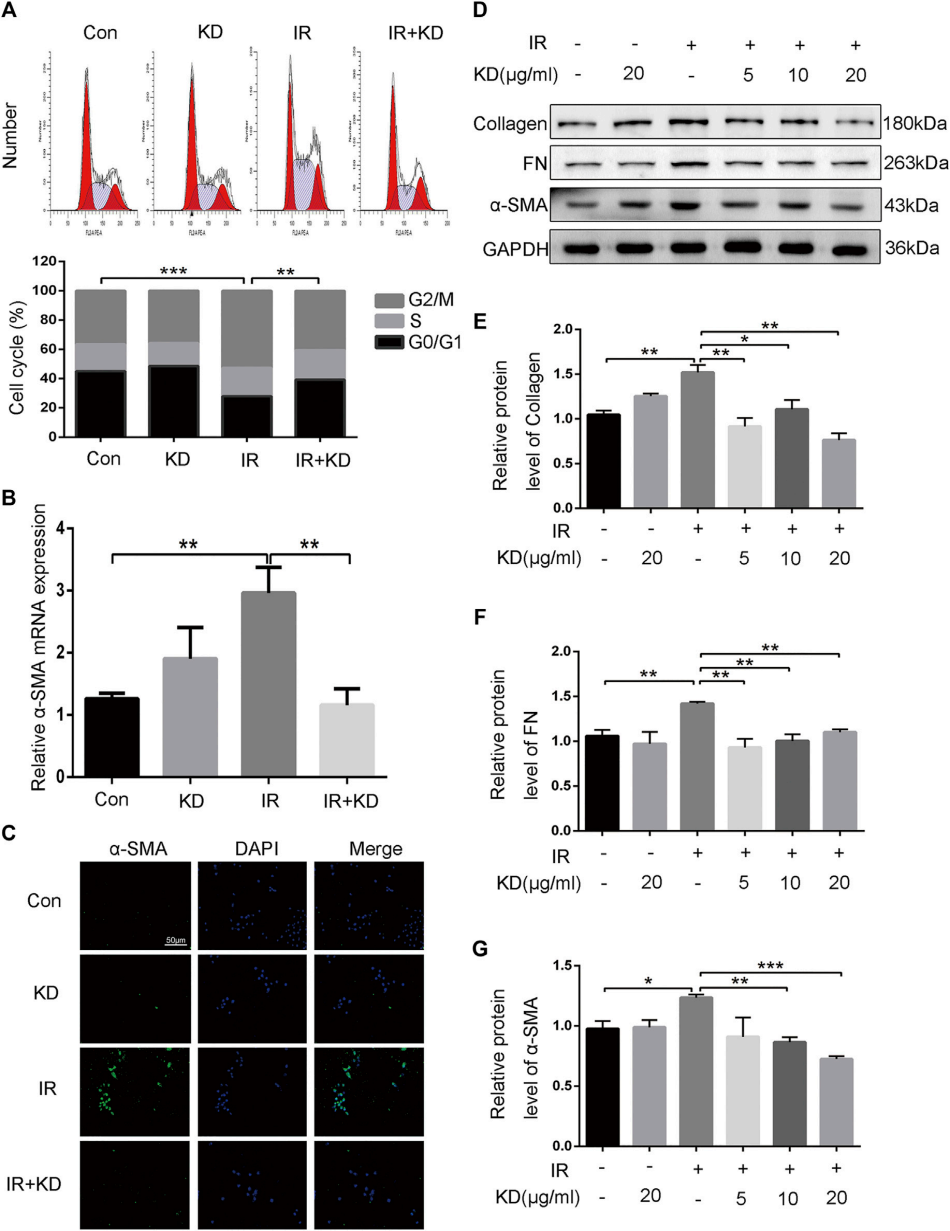
KD inhibits the proliferation of HSCs and the key proteins that promote the RILF **(A)** The results of the cell cycle showed that in the IR + KD group, the cell cycle arrested in the G0/G1 phase compared with the IR group, which indicated the inhibition of the cell proliferation **(B)** The expression of *a*-SMA mRNA in HSC-T6 cells increased after 6Gy irradiation, while the administration of KD after irradiation inhibited this expression **(C)** The protein fluorescence intensity of *α*-SMA increased in the IR group compared with the control groups, and the fluorescence intensity decreased in the IR + KD group compared with the IR group **(D-G)** Unirradiated HSC-T6 cells expressed little or did not express fibrosis-related proteins such as collagen, FN and *a*-SMA protein. The expression of these fibrosis-related proteins increased after 6Gy irradiation, and the administration of KD after irradiation inhibited the expression of these proteins. For all results in this figure, mean ± SEM. n. s. denotes not significant; *p < 0.05, **p < 0.01, ***p < 0.001.

Studies have shown that HSCs are the critical effector cells in the process of liver fibrosis (Wan et al., 2017; Wu et al., 2018a; Dewidar et al., 2019b). In order to study the regulatory effect of KD on the HSCs, we separately detected the cell cycle of each group and found that compared with the IR group, the HSC-T6 cells in the IR + KD group were blocked in the G0/G1 phase (Figure 4A), indicating that KD played a role in inhibiting the proliferation of HSCs. We detected the expression of *α*-SMA gene in each group, and the results showed that radiation exposure caused the activation of HSC-T6 cells at the transcription level, while the administration of KD inhibited this activation (Figure 4B). Furthermore, the expression of *α*-SMA protein in each group was detected by immunofluorescence. The results showed that the fluorescence intensity of *α*-SMA protein in the IR group was significantly higher than that in the control groups. While the fluorescence intensity of the protein in the IR + KD group decreased significantly, compared with the IR group (Figure 4C), indicating that the radiation exposure activated the HSC-T6 cells, while KD administration after irradiation reduced this activation. Subsequently, we tested the marker proteins of RILF. As shown in the Figures, the expression of the marker proteins decreased in the IR + KD group, compared with the IR group (Figures 4D–G). These results indicated that HSCs were activated after irradiation and produced proteins to promote fibrosis, while KD could inhibit the activation of HSCs and inhibit the production of these key fibrosis proteins.

### Screening of the Target Gene Connective Tissue Growth Factor and TGF-*β*1 Signaling Pathway

**FIGURE 5:**
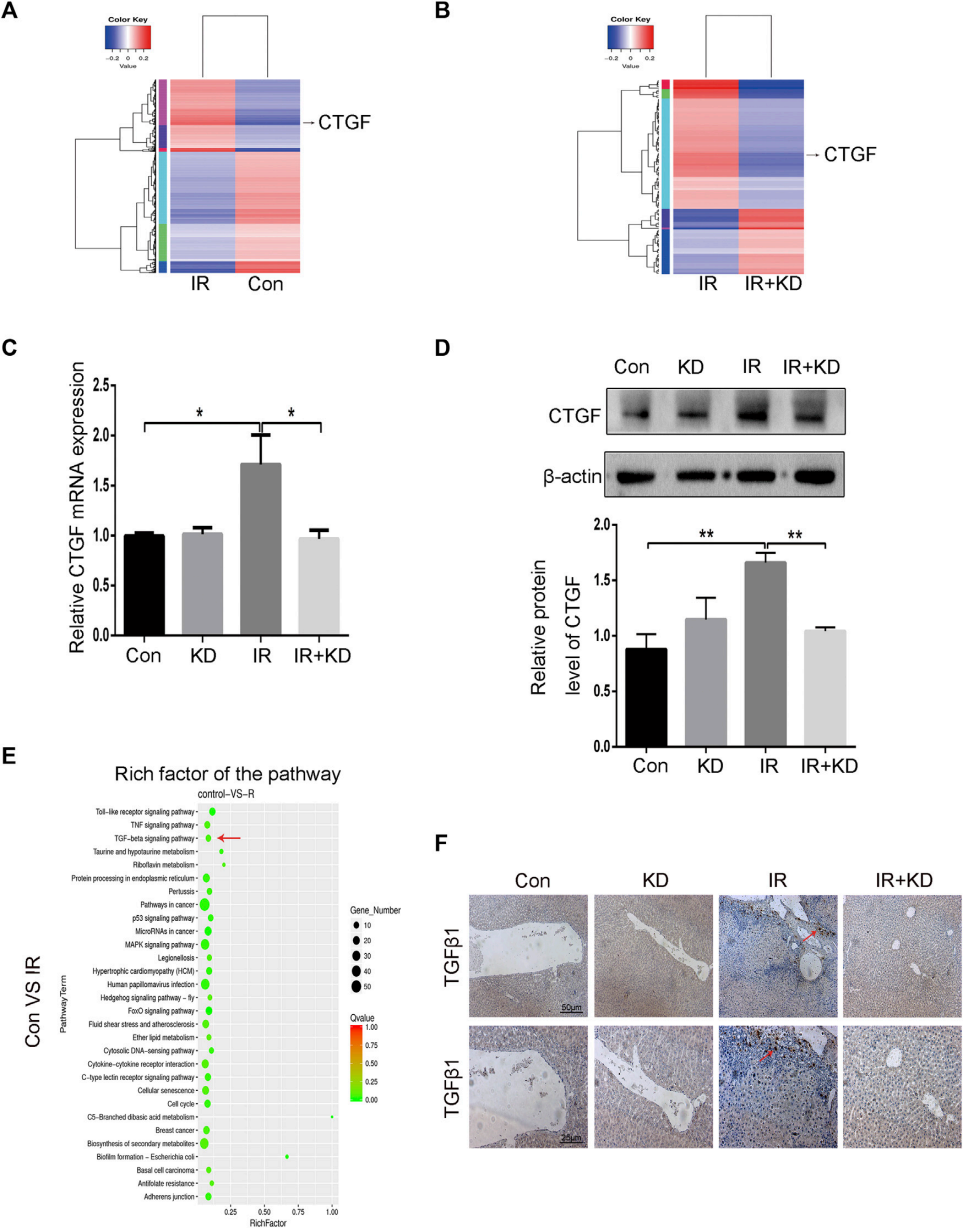
The key fibrosis-related gene and signaling pathway that are inhibited by KD **(A)** The results of high-throughput sequencing of the transcriptome showed that the expression of CTGF gene increased after irradiation **(B)** The results of high-throughput sequencing of the transcriptome showed that the expression of CTGF gene decreased after the administration of KD **(C)** The results of Real-Time PCR were consistent with the results of the sequencing. The expression of CTGF mRNA increased in the IR group, but decreased in the IR + KD group **(D)** The results of western blotting were consistent with the results of sequencing. The expression of CTGF protein increased in the IR group, but decreased in the IR + KD group **(E)** In the transcriptome KEGG gene pathway enrichment map, the TGF-β pathway was closely related to CTGF and RILF **(F)** The results of tissue TGF-β1 immunohistochemistry indicated that in the IR + KD group the expression of TGF-β1 in the liver tissue decreased compared with the IR group. For all results in this figure, original magnification, ×40 and ×100. Mean ± SEM. *p < 0.05, **p < 0.01.

To find the differential genes that KD targeted in the process of alleviating RILF, we performed transcriptome high-throughput sequencing. Through the differential gene screening and the cluster map analysis, we found that in the IR group, CTGF was a high-expressed gene compared to the control group, while in the IR + KD group, CTGF was low-expressed compared to the IR group (Figures 5A,B), indicating that CTGF might be an important target gene for KD to attenuate RILF. CTGF is an important fibrosis-promoting mediator downstream target of TGF-*β* that plays a key role in the liver fibrosis (Weiskirchen, 2016; Wu et al., 2018b; Alatas et al., 2020). Next, we verified the transcriptome sequencing results by Real-Time PCR and western blot analyses. It was found that CTGF was significantly upregulated both in the mRNA and protein levels after HSC-T6 cells irradiated with 6Gy rays, while the expression of CTGF decreased in the IR + KD group, compared with that in the IR group (Figures 5C,D). These results suggested that KD could inhibit the expression of CTGF in HSCs after irradiation.

The signaling pathway enrichment was conducted to screen the pathways upstream of CTGF that played a key role in the process of RILF. The bubble chart of the transcriptome showed a series of enriched signal pathways, among which the TGF-β pathway was closely related to CTGF and the extracellular environment (Figure 5E). Based on the results, we speculated that TGF-β pathway was involved in the process that KD alleviated RILF. In order to further verify this, we performed TGF-β immunohistochemical staining on the rat liver tissue. As shown in Figure 5F, expression of TGF-*β*1 increased in the IR group, while there was almost no expression of TGF-*β*1 in the IR + KD group. These results indicated that the TGF-*β*1 pathway played a key role in promoting liver fibrosis caused by radiation, and KD administration after irradiation could reduce the expression of TGF-*β*1.

### Kinsenoside Reduces Radiation-Induced Liver Fibrosis by Blocking TGF-*β*1/Smad/Connective Tissue Growth Factor Pathway

**FIGURE 6:**
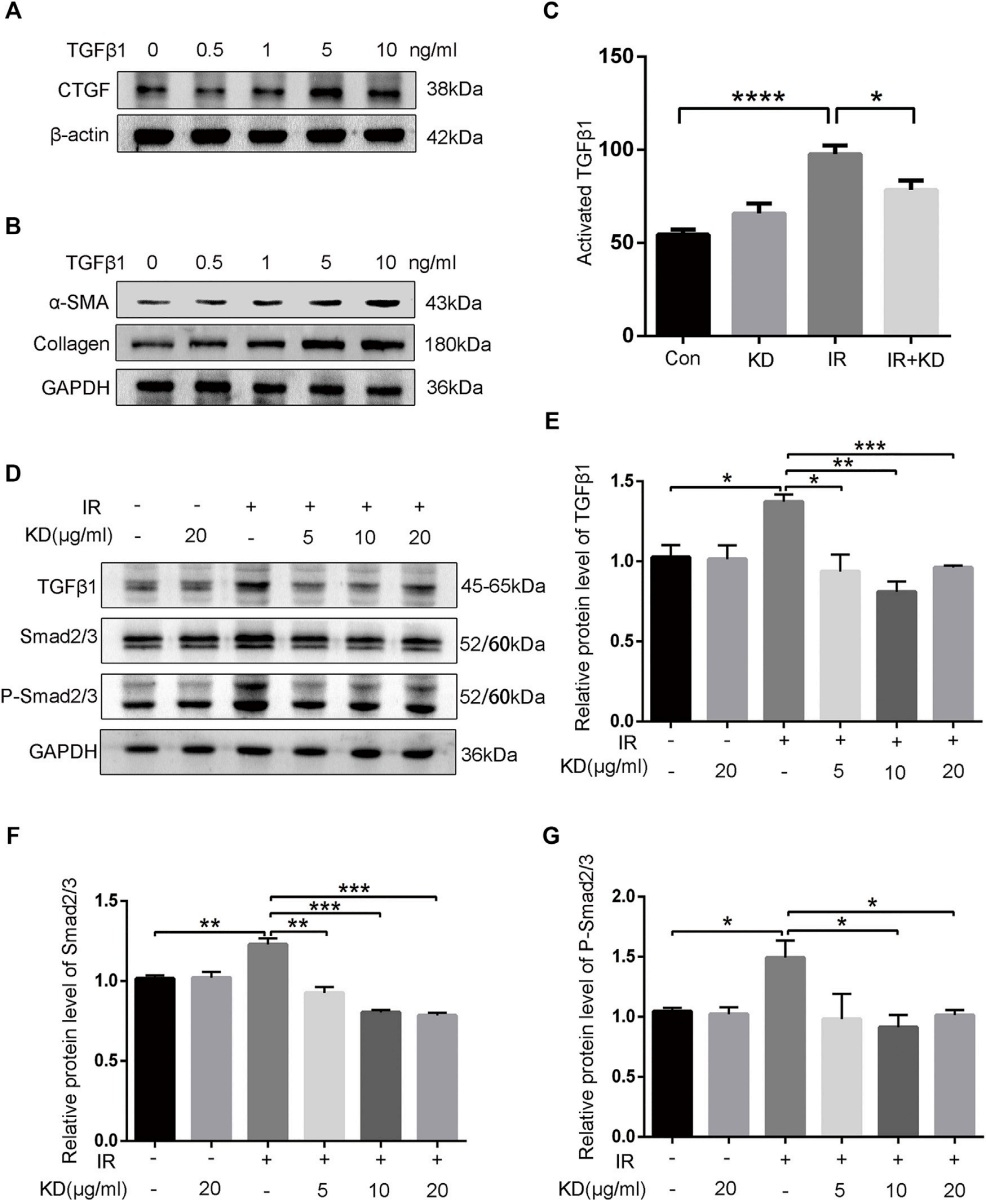
KD ameliorates the RILF by inhibiting TGF-β1/Smad/CTGF pathway **(A)** Exogenous TGF-β1 led to the increased expression of CTGF protein **(B)** Exogenous TGF-β1 induced an increase in the expression of collagen and *a*-SMA proteins **(C)** The TGF-β1 activity in the conditioned media significantly increased after 6Gy irradiation compared with the control group, while the TGF-β1 activity in the conditioned media in the IR + KD group decreased compared with the IR group **(D-G)** The expressions of TGF-β1 and downstream Smad2/3 as well as phosphorylated Smad2/3 reduced in the IR + KD group compared with the IR group. Mean ± SEM. *p < 0.05, **p < 0.01, ***p < 0.001, ****p < 0.0001.

**FIGURE 7:**
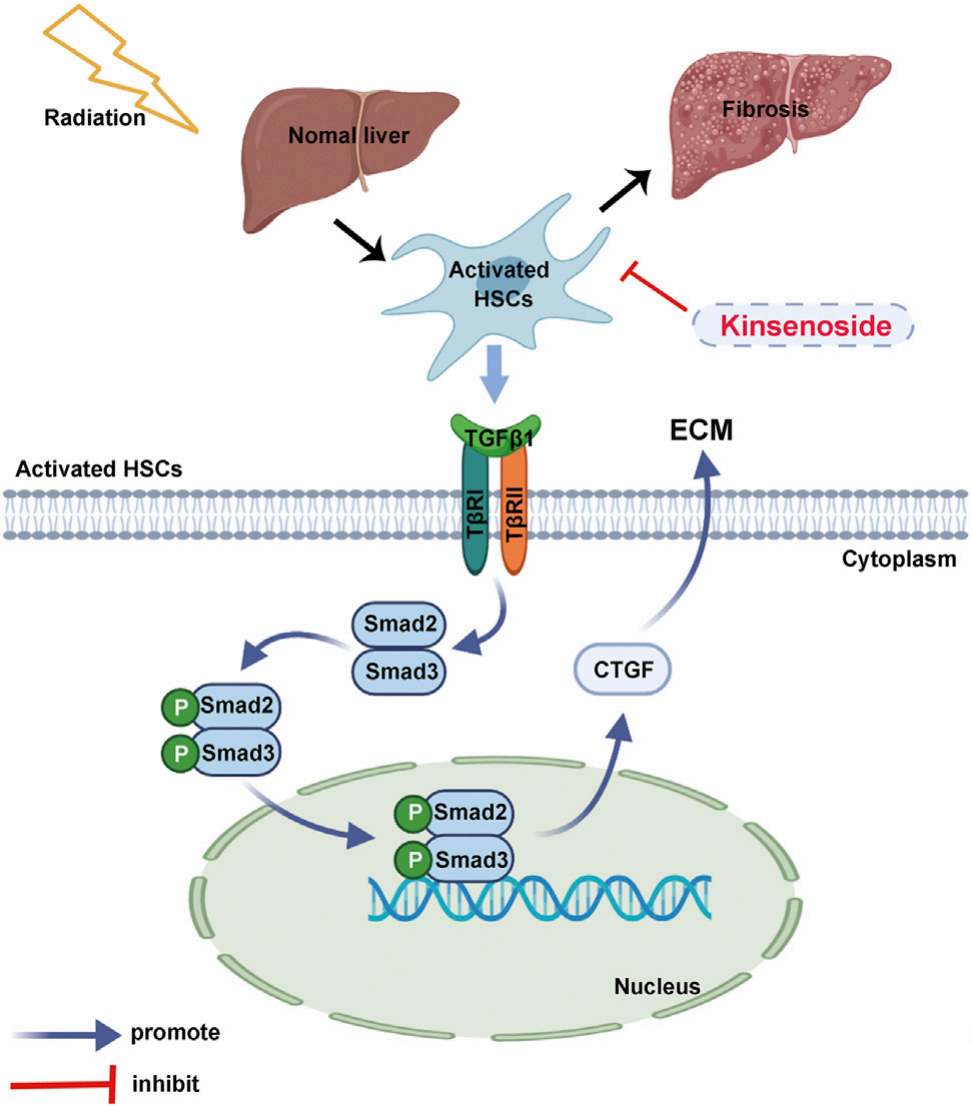
The schematic diagram. KD ameliorated the RILF through the regulation of the key molecule CTGF and TGF-β1/Smad/CTGF pathway in the HSCs.

To further clarify whether TGF-*β*1 promoted the activation of HSCs and the expression of fibrosis-related proteins, we detected the expression of fibrosis-related proteins after exogenous TGF-*β*1 acted on the HSC-T6 cells. As the concentration of exogenous TGF-*β*1 increased, the expression of CTGF protein increased, and the expression of collagen and *α*-SMA protein both showed a gradient increase (Figures 6A,B). Results showed that the increase in the concentration of TGF-*β*1 led to the activation of HSCs, which in turn increased the expression of CTGF protein and collagen, thereby producing the effect of promoting fibrosis. In addition, we collected cell supernatants from each group to test the content of activated TGF-*β*1 by the ELISA. The results showed that the secreted activated TGF-*β*1 was significantly higher in HSC-T6 cells after 6Gy irradiation than the control groups, while the secreted activated TGF-*β*1 decreased in the IR + KD group, compared with the IR group (Figure 6C). Next, we further analyzed the expression of TGF-*β*1 and its downstream pathway proteins. The expressions of TGF-*β*1 and its downstream proteins, total Smad2/3 and phosphorylated Smad2/3, were all increased after irradiation and decreased in the IR + KD group (Figures 6D–G). In addition, CTGF is the pro-fibrotic mediator downstream of the TGF-*β*1 pathway, and we have found that KD could inhibit the expression of CTGF. Based on these results, KD could inhibit the activated TGF-*β*1/Smad/CTGF pathway postirradiation, thereby inhibiting RILF (Figure 7).

## Discussion

The RILF is a kind of normal liver tissue damage induced by radiation, lacking effective prevention and treatment methods currently. As reported, the mortality rate of the radiation-induced liver injury in severe cases reaches 75%, and most of these patients eventually suffer from liver failure (Guha and Kavanagh 2011; Chen et al., 2015; Munoz-Schuffenegger et al., 2017). At present, the liver fibrosis is considered to be a healing response to the chronic liver injury. However, if the inducing factors of liver damage are not properly removed, the liver fibrosis will continue and cause serious distortion of the liver tissue structure, which may eventually lead to the liver failure and death (Aydın and Akçalı 2018; Roehlen et al., 2020; Kisseleva and Brenner 2021). In this study, we successfully established the rat model of the RILF, and the results of the HE and Masson staining indicated that KD significantly improved the RILF. In addition, we measured the fibrosis score and the content of hydroxyproline in the liver tissues of each group to further verify the effect of KD on alleviating the RILF. Our research showed that KD reduced the liver fibrosis in rats after radiotherapy, and provided a new method for the prevention and treatment of the RILF.

It’s well accepted that the RILF closely related to the continuous overexpression of a variety of inflammatory and fibrotic cytokines, but the underlying specific mechanisms remain to be undiscovered (Chen et al., 2017; Rosenbloom et al., 2017; Yuan et al., 2019b). The main cause of the liver fibrosis is the excessive accumulation of the fibrosis-related proteins such as collagen in the perisinusoidal space, and changes in these ECM components induce the hepatic sinusoidal endothelial cells (LSEC) to form the basement membranes, which interferes with the normal nutrient transport between blood and the surrounding cells, especially the liver cells, and ultimately leads to dysfunction (Natarajan et al., 2017; Ni et al., 2017). Based on the reported studies, we performed the immunohistochemical (IHC) staining of the livers and scored IHC scoring of the *α*-SMA protein, which was the marker of the liver fibrosis and the HSCs activation. The results showed that the *α*-SMA protein in the post-irradiated liver was downregulated when treated with KD. The results of the collagen immunofluorescence showed that the collagen expression in the IR + KD group was significantly lower than that in the IR group, which indicated the inhibitory effect of KD on the activation of the HSCs and the subsequent induction of the liver fibrosis. Additionally, we extracted the total protein of the liver tissue and detected the expression of the key proteins involved in the liver fibrosis. The results suggested that KD significantly reduced the expression of *α*-SMA and FN after radiotherapy.

KD, an extract of the traditional Chinese herbal plant *A. roxburghii*, has a variety of pharmacological effects, among which the liver-protecting effect shows significant importance (Xiang et al., 2016a; Xiang et al., 2016b). Studies have shown that KD can ameliorate the autoimmune hepatitis and protect against the CCl_4_-induced liver damage in mice, while the mechanism remains unknown (Xiang et al., 2016b). The results of our studies on the animal model shown that KD had protective effects on the RILF, which provided a theoretical basis for solving the urgent clinical problems on prevention and treatment of the RILF. Importantly, the drugs used to prevent the RILF are supposed not to affect the efficacy of radiotherapy on the malignant liver tumors. In this study, the xenograft tumors were inoculated and irradiated with a single 10Gy radiation after reaching the expected size. Subsequently, the nude mice were administered with KD, and the results showed that KD administration after radiotherapy did not affect the efficacy of tumor radiotherapy.

The HSCs are the main cells that produce the ECM in the damaged liver. Protein *α*-SMA acts as an important marker for the activation of the HSCs, and FN is an important ECM regulatory component in the fibrosis process related to the HSCs activation (Rygiel et al., 2008; Zollinger and Smith 2017; Klingberg et al., 2018). The activated HSCs migrate and accumulate in the tissue repairing site, then secrete a large amount of ECM and regulate ECM degradation (Chen et al., 2020). In addition, during the chronic liver injury, liver parenchymal cells are damaged, along with increasing of the activated HSCs and inflammatory cells (Weiskirchen and Tacke 2016). We found that the HSCs, the main effector cells of liver fibrosis, were activated by radiation and proliferated actively after 6Gy of radiation, and the expression of the fibrosis-related proteins significantly increased. The administration of KD effectively inhibited the activation and proliferation of the HSCs, and promoted a significant reduction in the expression of the fibrosis-related proteins.

CTGF is a kind of matrix protein that is commonly up-regulated in the liver fibrosis, which plays a key role in liver fibrosis (Weiskirchen, 2016; Wu et al., 2018b; Makino et al., 2018). Studies have shown that hepatocytes, bile duct cells, HSCs and many other cells express and secrete CTGF protein in the fibrotic liver (Williams et al., 2014; Ding et al., 2016; Ramazani et al., 2018). As reported, CTGF is significantly upregulated and plays a key role in the liver fibrosis (Xu et al., 2015; Tomei et al., 2016). Furthermore, CTGF is a downstream regulator of TGF-*β*1, which is the final link leading to the accumulation of ECM (Wu et al., 2018b). Various activating factors can promote the latent TGF-*β*1 turning into activated TGF-*β*1, and the latter binds to the corresponding receptor on the effector cells (Hinck et al., 2016). Growing evidence have shown that activated and overexpressed TGF-*β*1 eventually lead to the depletion of parenchymal cells and excessive tissue fibrosis (Moses et al., 2016; Liu et al., 2019; Wang et al., 2019). In this study, we screened the key target gene regulated by KD through high-throughput sequencing of the transcriptome, and CTGF was found to be a key molecule in the RILF. The signaling pathway enrichment suggested the importance of TGF-*β* signaling pathway in regulating the RILF, and the IHC staining of liver tissue showed the reduced expression of TGF-*β*1 in the IR + KD group. Our data showed that the irradiation activated and upregulated TGF-*β*1, which stimulated the HSCs, resulting in the increased expression of the fibrosis-related proteins. Furthermore, treatment with KD reduced the activated TGF-*β*1 after radiotherapy. Taken together, we figured out that KD alleviated the RILF via inhibition of the TGF-*β*1/Smad/CTGF axis.

This study has several limitations. Firstly, we lacked continuous monitoring at different time points before 24 weeks. Secondly, we failed to use the imaging methods to assess the degree of liver fibrosis in this study. In the follow-up researches, the imaging detection will be used to further verify the experimental results. Thirdly, the intermolecular interaction in the signaling pathway needs to be detailed.

In summary, this study discovered that KD could ameliorate the RILF through the regulation of the key molecule CTGF and TGF-*β*1/Smad/CTGF pathway in the animal model and the cell-level experiments. Additionally, KD showed no adverse effects on the tumor radiotherapy. These results have provided supporting evidence for the clinical application of KD as an innovative drug for the treatment of the RILF, and provided a basis for the use of reagents targeting the TGF-*β*1 pathway and CTGF molecule to prevent and treat the RILF.

## Data Availability

The datasets presented in this study can be found in online repositories. The name of the repository and accession number can be found below: National Center for Biotechnology Information (NCBI) BioProject, https://www.ncbi.nlm.nih.gov/bioproject/, PRJNA784416.
